# Wirelessly Actuated Thermo- and Magneto-Responsive Soft Bimorph Materials with Programmable Shape-Morphing

**DOI:** 10.1002/adma.202100336

**Published:** 2021-05-28

**Authors:** Jiachen Zhang, Yubing Guo, Wenqi Hu, Metin Sitti

**Affiliations:** Physical Intelligence Department, Max Planck Institute for Intelligent Systems, 70569 Stuttgart, Germany; Physical Intelligence Department, Max Planck Institute for Intelligent Systems, 70569 Stuttgart, Germany; Institute for Biomedical Engineering, ETH Zürich, Zürich 8092, Switzerland; School of Medicine and College of Engineering, Koç University, Istanbul 34450, Turkey

**Keywords:** active soft materials, liquid crystal elastomers, magnetic-responsive elastomers, programmable shape-morphing, soft robotics

## Abstract

Soft materials that respond to wireless external stimuli are referred to as “smart” materials due to their promising potential in real-world actuation and sensing applications in robotics, microfluidics, and bioengineering. Recent years have witnessed a burst of these stimuli-responsive materials and their preliminary applications. However, their further advancement demands more versatility, configurability, and adaptability to deliver their promised benefits. Here, a dual-stimuli-responsive soft bimorph material with three configurations that enable complex programmable 3D shape-morphing is presented. The material consists of liquid crystal elastomers (LCEs) and magnetic-responsive elastomers (MREs) via facile fabrication that orthogonally integrates their respective stimuli-responsiveness without detrimentally altering their properties. The material offers an unprecedented wide design space and abundant degree-of-freedoms (DoFs) due to the LCE's programmable director field, the MRE's programmable magnetization profile, and diverse geometric configurations. It responds to wireless stimuli of the controlled magnetic field and environmental temperature. Its dual-responsiveness allows the independent control of different DoFs for complex shape-morphing behaviors with anisotropic material properties. A diverse set of in situ reconfigurable shape-morphing and an environment-aware untethered miniature 12-legged robot capable of locomotion and self-gripping are demonstrated. Such material can provide solutions for the development of future soft robotic and other functional devices.

## 1 Introduction

Wirelessly actuated and remotely controlled active soft materials have captured significant research attention due to their promising potential applications in a large variety of fields with radically improved performance compared with traditional smart materials.^[1–5]^ These synthetic materials respond to environmental stimuli and exhibit capabilities that mimic or match the behavior or phenomena observed in nature.^[6–8]^ Among these smart materials, mechanically stimuli-responsive materials harvest energy from environmental inputs, e.g., light,^[9–11]^ heat,^[12,13]^ solvents,^[14,15]^ and physical fields,^[16–18]^ and convert it into mechanical energy for shape-morphing, without being encumbered by on-board power sources. These wirelessly powered materials could accomplish various functionalities, such as locomotion^[19–21]^ and object manipulation and transportation^[22–24]^ as actuators and sensors.

Among the abundance of active smart materials reported to date, liquid crystal elastomers (LCEs) and magnetic responsive elastomers (MREs) stand out from others recently, owing to their unique characteristics and distinctive merits. LCEs exhibit large strains (up to 400%) and high work density in response to multiple environmental stimuli, such as temperature,^[25–27]^ light,^[11,28]^ and electric field.^[17,18,29]^ The pre-defined alignment of the mesogens within LCEs, described by a director field $\vec{\text{n}}$ enables sophisticated 3D reversible shape-morphing that has been used in soft actuators and bio-inspired devices.^[6,11,30]^ External stimuli change the order parameter of the aligned mesogens, inducing contractile and tensile strains depending on the local director fields of LCEs. These local strains work in concert to achieve the designated shape-morphing behavior, which is often out-of-plane bending.

On the other hand, MREs consist of a soft elastomer (SE) matrix with embedded hard magnetic micro- or nanoparticles (MMPs or MNPs). External magnetic field generates local forces and torques on the embedded MMPs or MNPs. The distributed torques cause body deformation and a net rotation of an MRE material, while the forces experienced by the particles converge into a net force that displaces the MRE or deforms it.^[31]^ Magnetic actuation has the advantages of long-range, robust and fast actuation, and the capability of instantaneously and simultaneously exerting both force and torque on the same object.^[32]^ Besides, electromagnetic coil systems and permanent magnets can easily control the direction, strength, and spatiotemporal variance of a 3D magnetic field. This wide range of tunability of these control parameters endows MREs with unparallel versatility in exhibiting multiple different complex shape-morphing behaviors from a single piece of material,^[1,33–35]^ while most other active materials can only exhibit a single simpler deformation profile.^[36,37]^ With favorable controllability and versatility, MREs have been widely utilized in soft robots and other devices.^[34,38–41^]

As the search for more versatile and functional smart materials continues, the integration of different advantages of multiple active components into a single material becomes promising, via layered^[42–44]^ and monolithic forms.^[45–47]^ Attempts have been made to integrate LCEs with MREs for enhanced material properties.^[42,47]^ These previous studies represent a promising start of the exploration of multiresponsive smart materials for complex real-world applications. But the results shown so far are limited in versatility and adaptability, especially when anisotropic material properties are desired. Specifically, the existing bilayer approaches^[42]^ only showed proof-of-concept demonstrations with a simple and intuitive addition of the respective stimuli-responsiveness of LCEs and MREs via a bilayer or being glued together at the end. The full versatility of this approach remains unexplored. Alternatively, the monolithic approach represents a homogenous way of adding magnetic responsiveness to LCEs.^[47]^ But the monolithic material lacks anisotropy in terms of material properties due to the uniform distribution of the MMPs. Moreover, the concentration of MMPs is limited because an excessive presence of MMPs will hinder the director field of LCEs and impair its stimuli-responsiveness.

This work presents a multiresponsive soft bimorph material created via a facile fabrication approach with complex programmable 3D shape-morphing. This material orthogonally integrates the respective stimuli-responsiveness of LCEs and MREs for enhanced versatility and abundant degree-of-freedoms (DoFs) in control. It offers an advantage of integrating LCEs and MREs via various configurations for expanded design space. These variable configurations enable anisotropic material properties within individual samples with a two orders-of-magnitude difference and complex programmable 3D shape-morphing. The fully uncoupled inputs offer additional DoFs in activating distinct parts of the material to achieve different functionalities. In contrast to the monolithic approach reported earlier,^[47]^ this bimorph material exceeds in material heterogeneity and local addressability within a single device. These advantages of the bimorph material make it a promising candidate for future multifunctional actuators and sensors in diverse real-world applications. For example, biomedical tasks, such as minimally invasive surgery and targeted drug delivery, and environmental monitoring and remediation tasks could benefit from the extended design space and local addressability of the reported material.

## 2 Material Design Concept

**Figure 1:**
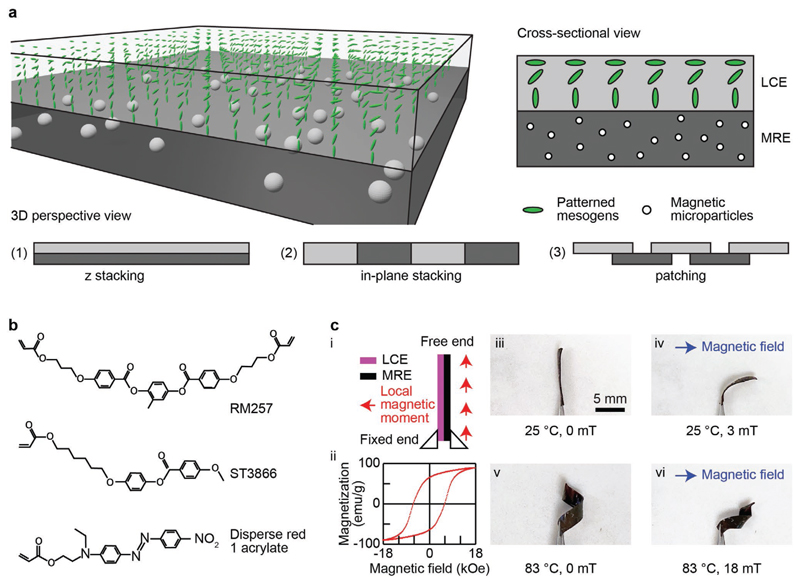
Conceptual illustration and experimental demonstration of the bimorph material with various configurations. a) A 3D and cross-sectional schematics illustrate the concept of the material. The liquid crystal elastomers (LCEs) and magnetic responsive elastomers (MREs) are integrated together via one of three configurations: *z* stacking, in-plane stacking, and patching. b) The chemical structures of the LCEs. c) Experimental demonstration of the multiresponsiveness of a sample made by the bimorph material via *z* stacking in response to temperature rise, magnetic field, and both. The sample exhibited in situ reconfigurable shape-morphing behaviors, resulting from the enhanced versatility with an additional degree of freedom (DoF) in actuation and control.

The concept of the reported material is schematically illustrated in Figure 1a, together with three distinct integration configurations of LCEs and MREs. LCEs and MREs are integrated via stacking across the thickness, within a plane, or only at small overlapping regions, which are hereafter referred to as “*z* stacking,” “in-plane stacking,” and “patching,” respectively. The three configurations offer unprecedented versatility and freedom in designing and utilizing the material to achieve different material properties, stimuli-responsiveness, and functionalities.

The chemical structures of the materials to form the LCEs are presented in Figure 1b. The LCEs possess a splay director field, which causes out-of-plane bending in response to external stimuli, such as temperature changes and UV light exposure. The MREs were made of an SE matrix (Ecoflex 0030, part A and B mixed at 1:1 mass ratio, Smooth-On) with embedded hard MMPs (NdFeB, MQP-15-7, Magnequench, an average diameter of 5 μm) at a designated mass ratio, e.g., 2:1, with a corresponding volume ratio of MMPs, e.g., 6.6% for the 2:1 mass ratio. Details of the preparation of the material are available in the Experimental Section.

The employed MMPs are selected because of their strong ferromagnetic properties and high Curie temperature (e.g., >300 °C). Within the working temperature range of this work, i.e., from room temperature to around 100 °C, the magnetization loss of these MMPs is negligible. Pure LCE has a certain degree of hysteresis in shape-morphing due to slight plastic distortion. For example, a small residual bending is often observed after the stimuli were removed. Nevertheless, the reported bimorph material exhibits much smaller residual deformation especially in the z stacking configuration, thanks to the elasticity of MRE.

## 3 Dual-Responsiveness of the Bimorph Material

The bimorph material orthogonally integrates the different stimuli-responsiveness of LCEs and MREs, which could be activated and controlled separately as well as conjunctly. A sample of z stacking experimentally demonstrated the resultant dual-stimuli-responsiveness of the material, whose results are shown in Figure 1c. The sample has a geometric dimension of 10 × 2 × 0.26 mm^3^ and consists of an LCE layer and an MRE layer of 0.04 and 0.22 mm thickness, respectively.

The sample was magnetized to program a uniform magnetization profile along its length (Figure 1c-i). The magnetic properties of the MMPs were characterized using a vibrating sample magnetometer (VSM) and results are given in Figure 1c-ii. The MMPs exhibit hard magnetic property, featuring a high remanence (the positive y-intercept of the curve) and coercivity value (the negative *x*-intercept of the curve). In other words, the MMPs can have programmed permanent magnetization direction and magnitude and possess strong magnetic responsiveness that enables the material to respond to relatively weak magnetic fields. Details of the sample fabrication and magnetizing process are available in the Experimental Section.

A tweezer fixed one end of the sample on a hot plate. The sample remained flat in the air at room temperature in the absence of external magnetic fields (Figure 1c-iii). When a magnetic field (3 mT) was applied, the MRE part of the sample experienced magnetic torques and forces according to the following equations

(1) $$\vec{\tau }=\vec{m}\times \vec{B}$$

(2) $$\vec{F}=\nabla \left(\vec{m}\cdot \vec{B}\right)$$

where $\vec{\tau },\vec{m},\vec{B},\;\text{and}\;\vec{F}$ are the torque, the magnetic moment, the magnetic flux density, and the force, respectively. As a result, the sample bent toward the direction of the magnetic field, trying to align its local magnetic moments with the magnetic field direction (Figure 1c-iv).

When the ambient temperature rose (83 °C), the sample formed a helical shape caused by the local contraction along its director field and the local expansion perpendicular to its director field. This phenomenon happened due to the reduced order parameter of its LCE part induced by the elevated temperature (Figure 1c-v). Lastly, when both stimuli were present, the sample curled into a helical shape while also bent toward the direction of the magnetic field (Figure 1c-vi). This demonstration proves that the integration of LCEs and MREs offers an additional DoF in activating and controlling the material behaviors and in situ reconfigurable shapes of the same sample. This additional DoF in control is valuable as smart active materials are being increasingly employed in devices that demand sophisticated functionalities to work in complex, realistic environments. Thus, the bimorph material exhibits better versatility than MREs alone, which is acknowledged as a promising and versatile smart material that has been utilized in a wide variety of devices and robots.^[5,23,40]^

## 4 Integration of LCEs and MREs

The reported bimorph material integrates LCEs and MREs via a facile and intuitive fabrication process. Uncured MREs were cast into negative molds with designed geometries. The LCE film, which had been cut into the designed shape, was placed upon the uncured MREs. A stable bonding was formed between LCEs and MREs during the curing process of MREs. No additional adhesive or adhesion layer is required. This facile and intuitive fabrication process has the potential to be scaled up in the future. The strong bonding between LCEs and MREs survives repetitive cycles of exerting and removing external stimuli such as magnetic field, temperature change, and UV exposure. No fatigue or delamination of the material was observed in experiments. Experimental characterization results of the material's durability are provided in the next section.

**Figure 2:**
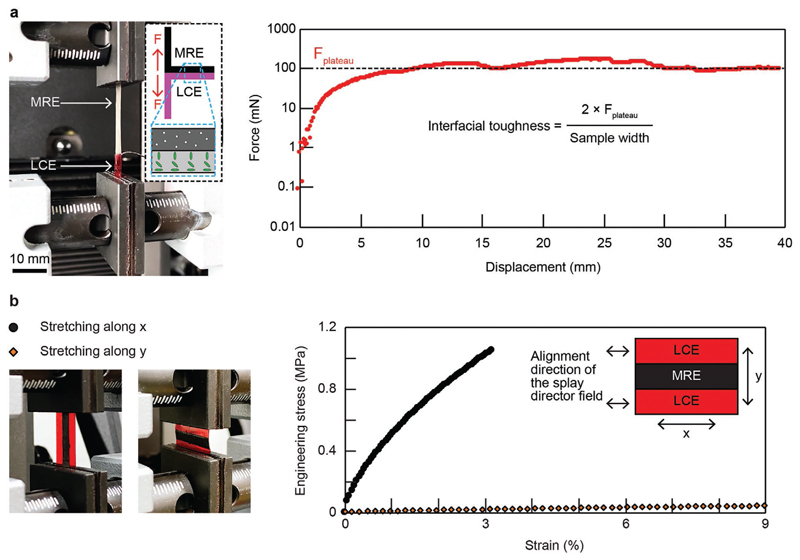
Characterizations of the bonding strength and the anisotropic material properties of the bimorph material. a) Characterization of the bonding strength between the LCE and MRE layers of a sample with the *z* stacking configuration in a 180° peeling test. b) Tensile characterization of the material property along two orthogonal axes of a sample with the in-plane stacking configuration. Results demonstrate the material has anisotropic material properties with a two order-of-magnitude difference.

A 180° peeling test characterized the bonding strength of the bimorph material using a sample with the *z* stacking configuration and a geometric dimension of 20 × 7.1 × 0.22 mm^3^ with a 0.04 mm thick LCE layer and a 0.18 mm thickness MRE layer. Experimental results suggest an interfacial toughness of about 28 J m^−2^. The experimental setup, including the relative alignment of the LCE director field with respect to the peeling direction, and the results are shown in Figure 2a. Details of the sample fabrication process and the peeling test are available in the Experimental Section.

As mentioned above, we integrate LCEs and MREs with three configurations. The distinctive three configurations lead to versatility in the integration of LCEs and MREs, compared with monolithic and conventional bilayer approaches. In particular, the integration of the two components via an in-plane zebraic strip configuration, i.e., in-plane stacking, endows the material with anisotropic mechanical properties that are two order-of-magnitude different. Figure 2b presents the experimental characterization results of a sample of in-plane stacking with a geometric dimension of 17.4 × 9.0 × 0.20 mm^3^. Each strip has a width of 3.0 mm. The sample was stretched along its length *x* and width *y* separately. Results show a significant difference between their stress-strain curves, which correspond to Young's moduli of 37.6 and 0.478 MPa for the direction along *x* and *y*, respectively. Details of the tensile test are available in the Experimental Section. The tensile stiffness of the sample along its length *x* is nearly 80 times stronger than the one along its width *y,* suggesting a dramatically anisotropic material property.

## 5 Characterization of Stimuli-Responsiveness

**Figure 3:**
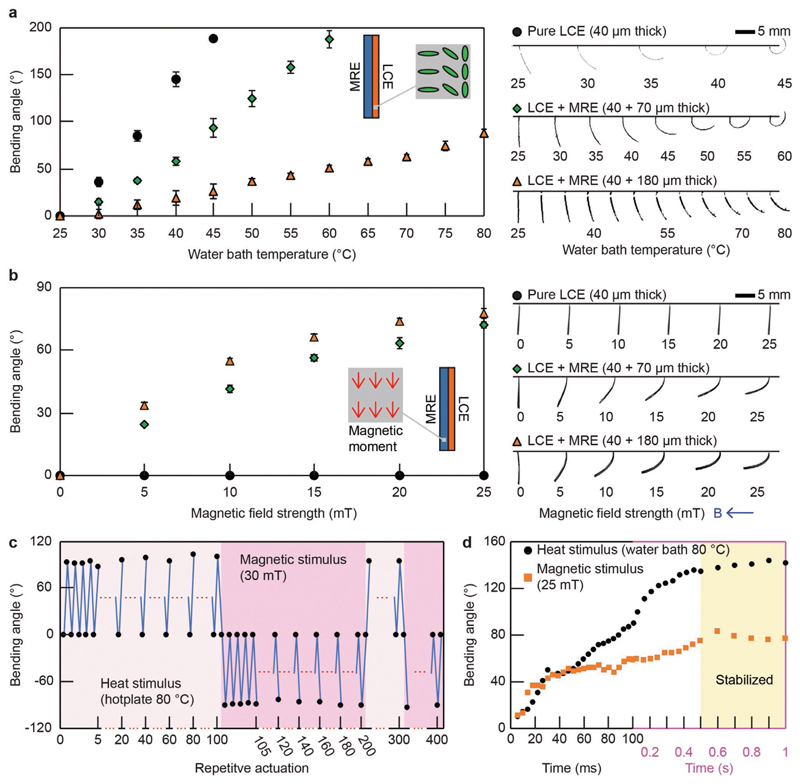
Experimental characterizations of the responsiveness of the bimorph material to temperature rise and external magnetic field strength. a) Samples of *z* stacking submerged in a water bath bent in response to ambient temperature rise. The bending angle of all samples increased with the temperature, while the response was weaker for samples with a thicker MRE layer. Each data point and error bar represent the average and the standard deviation of three measurements, respectively. Experimental photographs of the samples are presented on the right-hand side. b) Samples of *z* stacking in air reacted to the externally applied uniform magnetic field. The samples with an MRE layer bent with the magnetic field, while the sample without an MRE layer exhibited no response. Each data point and error bar represent the average and the standard deviation of three measurements, respectively. Experimental photographs of the samples are presented on the right-hand side. c) Response of a bimorph material in repetitive 400 actuation cycles of the heat and magnetic stimuli. d) Response speed of a bimorph material to the heat and magnetic stimuli.

The responsiveness of the bimorph material to temperature changes and magnetic fields was characterized using a sample of *z* stacking with a 2D geometric dimension of 8 × 2 mm^2^ and a varying thickness value of 40, 110, and 220 μm. The layer of LCE has a thickness of 40 μm while the layer of MRE has a thickness of 0, 70, and 180 μm, respectively. The sample was magnetized along its length. The sample was fixed by one end and submerged in a water bath placed on a hot plate. The water temperature was monitored and recorded, together with the corresponding out-of-plane bending angle of the sample. The results of the sample with different layer thickness of MRE are reported in Figure 3a. The sample responded to the temperature rise via out-of-plane bending in all cases. With the increase of the layer thickness of MRE, the bending response of the sample became weaker.

The samples were then placed in air in the presence of an externally applied uniform magnetic field with a varying strength. The magnetic field strength was monitored and recorded, together with the corresponding out-of-plane bending angle of the sample. The results of the samples with different layer thicknesses of MRE are reported in Figure 3b. It is obvious that the samples, except the one without an MRE layer, responded to the magnetic field via bending. And the bending angle increased with the field strength. With the increase of the layer thickness of MRE, the bending response of the sample to the magnetic field became stronger. Therefore, the ratio between bending amplitude from temperature and that from the magnetic field is controllable in a large range for different applications by carefully designing the LCE and MRE layer thickness and controlling the temperature and magnetic field. Especially, the *z* stacking material can be easily designed into thermo-responsive dominant or magneto-responsive dominant depending on the requirements of various applications.

The durability of the bimorph material was characterized in repetitive actuation cycles of heat and magnetic stimuli. A *z* stacking sample of 8 × 2 × 0.2 mm^3^ was prepared with a 40 μm thick LCE layer and a 160 μm thick MRE layer. The sample was magnetized along its length. It was repeatedly activated for 100 cycles using temperature rise (from room temperature at 25 to 80 °C by a hot plate) first and then using magnetic field (from 0 to 30 mT by a permanent magnet) for a total of 400 cycles. The resulting cyclic bending angles are shown in Figure 3c. No fatigue or delamination was observed, and the sample maintained a consistent stimuli-responsiveness during this cyclic test. Such result suggests high durability of the material and a strong bonding between the LCE and MRE layers in our reported bimorph material.

Both LCE and MRE exhibited fast responsiveness when exposed to stimuli. The reported bimorph material shows a slightly slower response speed to a single stimulus due to the presence of passive parts. For example, when a *z* stacking sample is activated by heat, the LCE part needs to deform not only itself but also the MRE layer, which is a passive part in this case. The response speed of the bimorph material was characterized using a z stacking sample of 8 × 2 × 0.2 mm^3^ with a 40 μm thick LCE layer and a 160 μm thick MRE layer. The sample was magnetized along its length. The bending angle of the sample was monitored when the sample was submerged in a water bath at 80 °C and placed within a magnetic field of 25 mT. Results are shown in Figure 3d. The sample took about 0.5 s to reach stable states in response to the stimuli.

## 6 Versatile, Reconfigurable Shape-Morphing Behaviors

**Figure 4:**
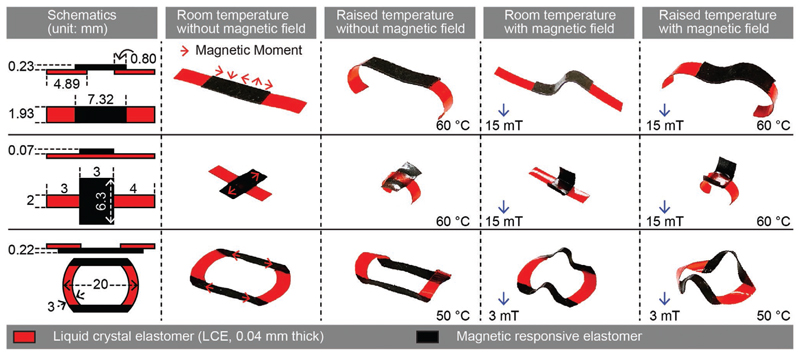
Complex dual-responsive shape-morphing behaviors of the bimorph material in response to the external temperature change and magnetic field, applied separated and concurrently. Three representative devices with in situ reconfigurable shape-morphing are shown in three separate rows. The first column of all rows contains schematic illustrations of the devices with given geometric dimensions. The second to fifth columns contain experimental photographs of the shape-morphing of the devices in different conditions: the second column shows their relaxed shapes at room temperature while no external magnetic field was present; the third, fourth, and fifth columns display their shape-morphing in response to ambient temperature rise, an externally applied magnetic field, and the simultaneous exertions of both stimuli of temperature rise and magnetic field, respectively. Dimensions in the schematics are not to scale for better visualization.

The versatility of integrating LCEs and MREs, especially via the patching configuration, results in a wide variety of possible complex shape-morphing behaviors. The decoupled multiple stimuli could be applied separately or conjunctly to induce diverse deformation from the same sample, enabling the in situ reconfigurability of the shape-morphing behavior. This section presents some representative 3D shape-morphing behaviors exhibited by the bimorph material to showcase its versatility and reconfigurability. Experimental photographs of samples (a strip, a cross, and a loop) in the relaxed state, activated by temperature rise, activated by the external magnetic field, and activated by both stimuli are shown in the second to the fifth column in Figure 4, whereas this first column contains a conceptual schematic of the sample with designed parameters. The samples were placed directly on the surface of a hot plate with its temperature set to the desired value. A permanent magnet was used to generate the actuation magnetic fields. From these exemplary demonstrations, we observed that LCEs and MREs in each sample could either be actuated separately by temperature rise and magnetic field or be actuated conjunctly.

The multiresponsiveness of the bimorph material offers an additional DoF in designing and actuating the samples into complex 3D shapes with the capability to in situ reconfigure the shape-morphing by adding or removing one of the stimuli. For example, four distinct shapes of the strip were achieved by manipulating temperature (high/low) and magnetic field (on/off). A reversible transition exists between any two of the four shapes shown in Figure 4, resulting in a total of six possible shape reconfigurations with the same sample via simply switching on or off the temperature and/or magnetic field inputs. For example, the shape of a sample could be changed from the one shown in the third column of Figure 4 to the one shown in the fifth column by simply adding a magnetic field to the workspace.

These enriched in situ reconfigurability could largely expand the functionalities of the bimorph material. Furthermore, depending on the patching design, shape morphing of LCEs and MREs can either be coupled (e.g., the strip) or be relatively decoupled (e.g., the cross and the loop), which provides more design freedom for the proposed bimorph materials. Finally, the above demonstrations used fixed temperatures and magnetic fields, which can be replaced by continuously changing the stimuli and optimized to enable other shape-morphing behaviors. The magnitude of the stimuli used here depends on the specific design of each device. For example, the loop deforms easier than the other two, thus the stimuli were set to be weaker than others to avoid clashing between different parts of its body.

## 7 Untethered Miniature Millipede-Like Robot with Locomotion and Self-Gripping

**Figure 5:**
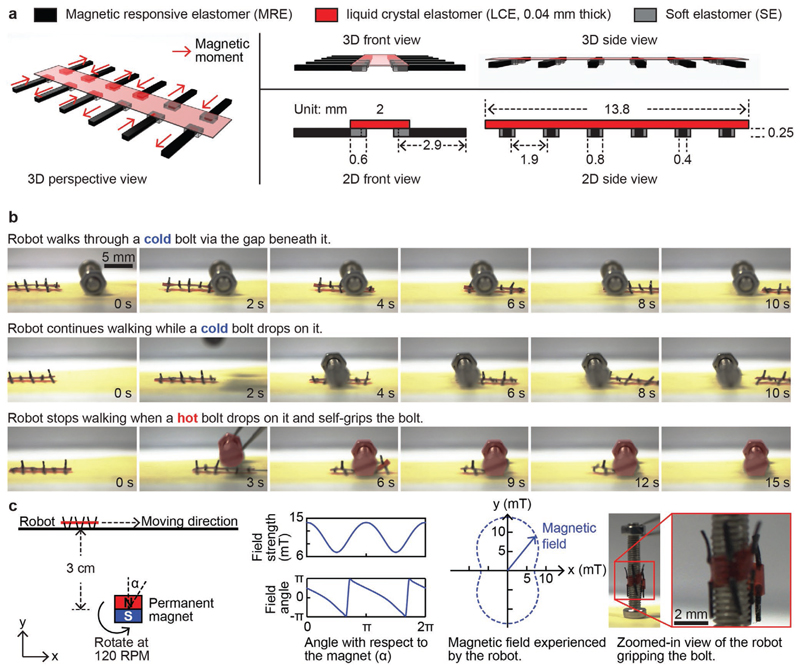
Experimental demonstrations of an untethered miniature 12-legged robot made of the bimorph material with capabilities of locomotion and self-gripping. a) Schematics illustrating the design and geometric features of the robot. The thickness of the LCE layer is exaggerated for better visibility. b) Snapshots of experimental videos captured by a side-view camera. The robot walked through the gap beneath a cold bolt (the first row), continued walking when a cold bolt dropped on it (the second row), and self-gripped the bold when a hot bolt dropped on it (the third row). The hold bolt was pseudo-colored with red for better visibility. c) Schematics illustrating the experimental setup of this experiment. The magnetic field cycling around the permanent magnet is drawn, together with the magnetic field experienced by the robot. A zoomed-in view of the robot gripping the hot bold is presented. The video of this experiment is available in Movie S1 in the Supporting Information.

This section reports an untethered mobile robot made by the bimorph material. The robot walked in a gait like a millipede on the surface of a substrate in air and in water under the actuation of a rotating magnetic field. It has 12 legs made of MRE and a central body made of LCE. And it self-gripped to a hot bolt upon contact. Schematics illustrating the design of the robot are shown in Figure 5a. The 12 legs of the robot bear local magnetic moments with alternating directions. In the presence of a global magnetic field, each leg bent to align its free end with the field direction. As the field rotated, the free end of each leg also rotated in synchronization, mimicking the movement of an oar. As a result, the leg lifted the robot up (bending downward), pushed the robot forward (bending backward), disengaged from the substrate (bending upward), moved back to the starting pose (bending forward), and repeated this periodical motion. Thanks to the alternating magnetic moment directions, there are always at least 6 legs supporting the robot at any time instance. Details of the leg motions are included in the Supporting Information.

Snapshots of the robots during experimental trials in different scenes are presented in Figure 5b. In the first scene, the robot walked through a positioned cold bolt via the gap beneath it. In the second scene, a cold bolt was dropped onto the robot while the robot was walking, and the robot continued walking without being disturbed. In the third scene, a hot bolt (with a surface temperature at around 100 °C) was dropped onto the robot while the robot was walking. The robot stopped walking and self-gripped the bolt by curling its body completely around the bolt.

During its magnetically actuated locomotion, its LCE part worked as a passive and suspensory section to hold all legs together and keep the body steady. Since LCE does not respond to magnetic field, having pure LCE as the central body avoids any undesired forces or torques being exerted on the body that could cause undesired tilting or twisting. A similar robot made of pure MRE was previously reported.^[35]^ However, that robot only worked in water or silicone oil, which lifted the bodyweight of the robot. And it had to bear closely packed antiparallel magnetic moments on its central body to alleviate unwanted magnetic torques because every part of the robot responded to the global magnetic field.

In comparison, the robot made of the bimorph material exhibits more reliable and effective locomotion in a more challenging environment, i.e., in air, as well as in water. Without the need to worry about the unwanted reaction of the central body, the robot is designed to possess an additional self-gripping capability. The robot self-grips a hot object that comes into its vicinity, showcasing its capability of environmental awareness. This kind of sensitivity to the environment is a widespread and useful feature in nature, such as the snap-through instability-based shape-morphing of a Venus flytrap that is triggered by a foreign object touching the flytrap sensory hairs. It could be utilized to enrich the catalog of functionalities of such miniature devices, such as remote sensing and autonomous cargo grasping. This capability of separately activating different parts of a robot leads to better controllability in robot behavior and functionality than conventional robots. Experimental videos of this robot walking in air and water, while responding to magnetic field, environmental temperature change, and UV exposure, are available in Movie S1 in the Supporting Information.

## 8 Conclusions

This work reports a bimorph material that combines the respective advantages of LCEs and MREs via a facile fabrication process to widen the material design space and enhance the control versatility. Through three configurations, the corresponding stimuli-responsiveness of the two active materials are orthogonally integrated into the bimorph material, which could be activated and controlled separately as well as conjunctly. The multiple configurations offer freedom and more DoFs in designing and actuating the materials to achieve complex 3D shape-morphing behaviors that could be in situ reconfigured. The facile fabrication process relies on soft lithography and mold-casting while not requiring an additional adhesion layer between LCE and MRE, which has the potential to be scaled up in the future for parallel and high-throughput mass production.

This work includes representative examples of these newly enabled deformation profiles to showcase the potential and capability of the bimorph material. Moreover, this work reports the design as well as experimental results of an untethered miniature robot that is capable of locomotion and self-gripping. In comparison with the existing literature, the facile and heterogenous integration of two stimuli-responsive materials in this work extends the design space for soft actuators, sensors, and robots in various applications and provides insights for the development of next-generation soft robotic and other functional devices. The uncoupled control of different parts of the material enables advanced functionalities that require complex material behaviors. This local addressability is especially useful in multifunctional devices, such as the demonstrated legged mobile robot with environmental awareness.

The LCE part of the bimorph material also responds to UV and visible light exposure via out-of-plane bending,^[11]^ which is not elaborated in this work due to its similarity with the heat-responsiveness. However, using UV light as the activation and control input instead of heat could have the advantages of improved spatiotemporal resolution and faster actuation. Thus, the activation and control methodology of the bimorph material via conjunctive employment of heat, magnetic field, and UV light for optimal performance will be investigated in the future.

## 9 Experimental Section

### LCEs

Two glass substrates were cleaned with deionized water, acetone, and isopropyl alcohol sequentially, and then spin-coated with PI2555 and SE1211 for planar and vertical surface alignment, respectively. A cloth was used to uni-directionally rub the PI2555-coated substrate for planar alignment of liquid crystal monomer along the rubbing direction. These two substrates were assembled together with UV-curable glue to form a liquid crystal cell with 40 μm cell thickness defined by spherical microparticles. A mixture of 65 wt% 4-methoxybenzoic acid 4-(6-acryloyloxy-hexyloxy)phenyl ester (ST3866, SYNTHON Chemicals), 32 wt% 1,4-bis[4-(3-acryloyloxypropyloxy)benzoyloxy]-2-methylbenzene (RM257, SYNTHON Chemicals), 2 wt% disperse red 1 acrylate (DR1A, Merck KGaA), and 1 wt% photoinitiator 2-benzyl-2-(dimethylamino)-4'-morpholinobutyrophenone (Merck KGaA) was melted on a 120 °C hot stage and mixed with a magnetic stirring bar for 1 h. The chemical structures of abovementioned materials are illustrated in Figure 1b. The melted mixtures were then filled into assembled liquid crystal cells through capillary attraction on a 120 °C hot stage to avoid flow alignment. The liquid crystal cells were then cooled to room temperature at a cooling rate of 60 °C per hour. The mixtures were then photopolymerized at room temperature with an UV lamp for 1 h. Finally, the polymerized LCE thin film was separated from glass substrates.

### SEs and MREs

Parts A and B of Ecoflex 0030 (Smooth-On) were mixed uniformly at a 1:1 mass ratio. To make MREs, this mixture was then mixed with MMPs (NdFeB, MQP-15-7, Magnequench, an average diameter of 5 μm) at a designated mass ratio, e.g., 2:1 (mixture: MMPs). The mixture was stirred and degassed to remove any trapped air. Then, the liquid mixture was casted into concave modes with a designed thickness to cure at room temperature for 3 h. Finally, the cured SE or MRE thin film was separated from the substrate.

### Bimorph Materials

The reported bimorph material with various integration configurations was prepared by placing LCEs in the modes prior to the casting of MREs or on top of the casted MREs. The curing of MREs formed a bond between LCEs and MREs. Devices were made of the material via laser cutting (ProtoLaser, LPKF Laser & Electronics AG) according to designs made in a software (AutoCAD, Autodesk Inc.). A magnetization profile was then programmed into the device by deforming it and magnetizing it in a strong magnetic field (1.8 T) generated by a VSM (EZ7, Microsense).

### Peeling and Tensile Tests

The sample was tested using a universal testing machine (Instron 5942, Instron, Norwood, MA, USA). The test was conducted with a constant peeling speed of 5 and 1 mm min^−1^ for the peeling and tensile tests, respectively. Interfacial toughness was calculated by timing the plateau force in the peeling test by two and then dividing it by the width of the sample.

### Generation of Stimuli

Heat stimulus was applied using a hot plate (Fisherbrand Isotemp) directly or through a water bath. Applying the heat stimulus using a water bath was uniform and accurate, while using the hot plate directly was faster. The response of the bimorph material on a hot plate was weaker than its response in a water bath due to the nonuniformity and heat convection. The magnetic stimulus was applied using the VSM or a permanent magnet.

## Data Availability

The data that support the findings of this study are available from the corresponding author upon reasonable request.
