# Supplementary material for: Wirelessly Actuated Thermo‐ and Magneto‐Responsive Soft Bimorph Materials with Programmable Shape‐Morphing
Source: Adv Mater. 2021 May 28;33(30):2100336. doi: 10.1002/adma.202100336 (PMC7612658; doi:10.1002/adma.202100336)
Supplement: Supplementary file 1 — Supporting Information [file ADMA-33-2100336-s001.pdf]

© 2021 Wiley-VCH GmbH

ADVANCED MATERIALS

Supporting Information

for *Adv. Mater.,* DOI: 10.1002/adma.202100336

# Wirelessly Actuated Thermo- and Magneto-Responsive Soft Bimorph Materials with Programmable Shape-Morphing

*Jiachen Zhang, Yubing Guo, Wenqi Hu, and Metin Sitti**

# Supporting Information

## Wirelessly actuated thermo- and magneto-responsive soft bimorph materials with programmable shape-morphing

Jiachen Zhang†, Yubing Guo†, Wenqi Hu†, and Metin Sitti*

† Equally contributing first authors
* Correspondence to: sitti@is.mpg.de

### S1. Fabrication process of the untethered mobile robot

The twelve legs of the robot were made by casting uncured MRE into negative molds with predefined in-plane geometries. The mold bearing the MRE was placed in a vacuum chamber and degassed to remove trapped air. After curing at the conditions specified by the manufacturer, the legs were taken out and magnetized via the process described in Experimental Section to program homogeneous magnetization profiles into them. Next, the magnetized legs were placed into a second negative mold with accommodating geometric features to fit the legs while leaving space for the connection pads. Uncured SE without embedded MMPs was cast into the mold to fill the space for the connection pads. Excess SE was removed by scraping using a razor blade. The mold was placed in a vacuum chamber and degassed to remove trapped air.

The central body of the robot was made by LCE, which was laser cut from an LCE film fabricated via the procedure described in Experimental Section. The central body of the robot was aligned and placed on top of the uncured SE, which has been cast into the second mold. After curing at the conditions specified by the manufacturer, the legs of the robot were connected to the central body via the connection pads. The robot was then taken out from the mold and ready to be used. The two molds utilized in this fabrication process and some important steps are schematically illustrated in **Figure S1**.

### S2. Leg motions of the untethered mobile robot

The twelve legs of the robot are made of MREs and bear homogeneous magnetization profiles along their length. Each leg is connected with the central LCE body of the robot via a connection pad formed by SE. Thus, each leg can be viewed as a cantilever beam with a fixed end and a free end. Once a rotating global magnetic field is applied, all the legs are driven by the magnetic field to bend and rotate along with the field direction. The twelve legs could be divided into two groups of the same size, within which the legs have magnetic moments in the same direction.

At any instance, the motions of legs from different groups have a fixed phase difference of 180° and symmetrically counteract each other to cancel out unwanted vibration, tilting, or direction shifting. Since the rotations of all legs are nonreciprocal and along the same direction, the robot moves forwards under the joint efforts of all twelve legs. Thanks to the symmetric design of the robot, the robot could also reverse its moving direction by reversing the rotating direction of the ambient magnetic field. The motion of individual legs and the overall walking behavior of the robot are schematically illustrated in **Figure S2**.

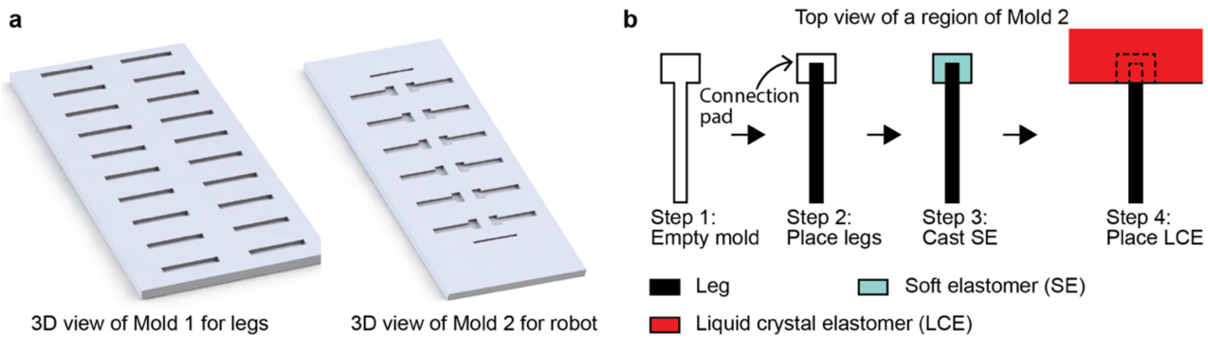


**Figure S1. Schematics illustrating the fabrication process of the reported miniature robot. a)** 3D renderings of the two molds used in the two stages of the robot fabrication. **b)** Series of top views of mold 2 showing the steps of placing the already cured legs into Mold 2, casting uncured SE, and aligning and placing the central robot body on top of the uncured SE.

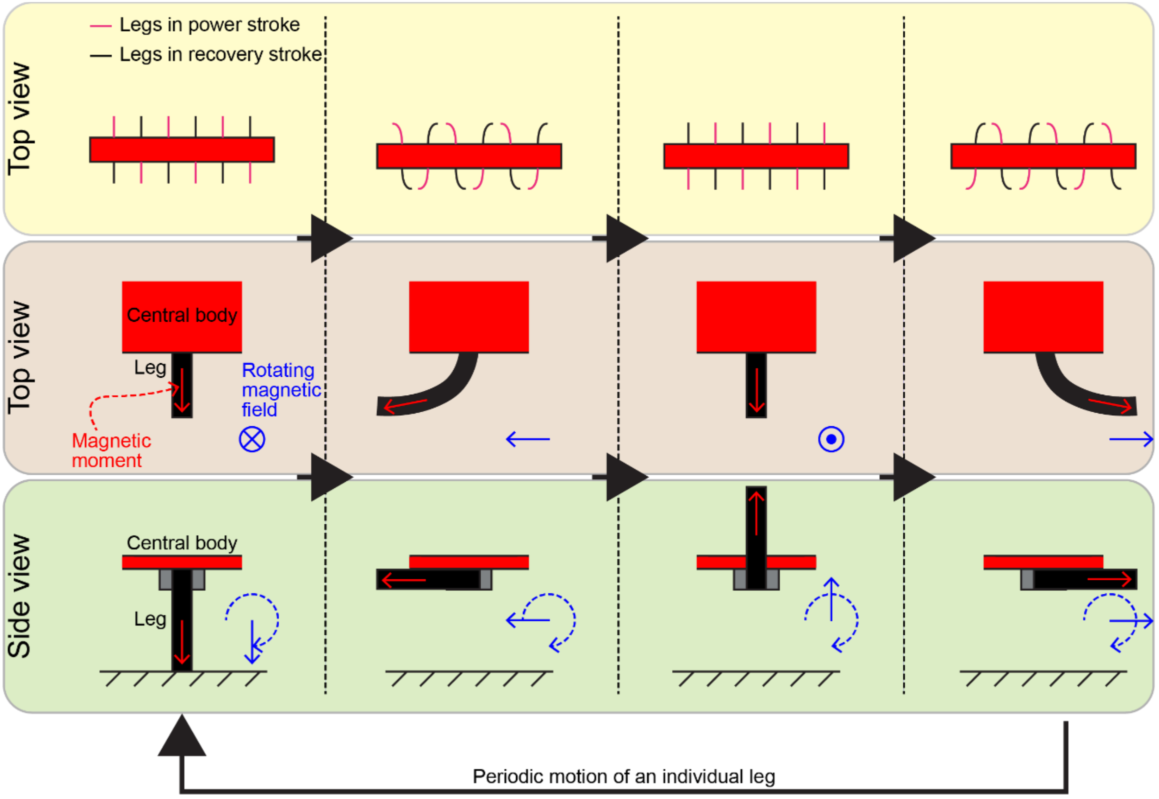


**Figure S2. Schematics illustrating the motions of individual legs of the robot and their overall behavior.**

## Supporting Movie Legend

**Experimental videos of an untethered miniature millipede-like robot exhibiting locomotion and self-gripping.** A rotating magnetic field served as the activation and control input. Segment 1: The robot walked on a flat substrate in the air and passed through the gap beneath a cold bolt. Segment 2: The robot walked on a flat substrate in the air and kept walking while a cold bolt dropped on it. Segment 3: The robots walked on a flat substrate in the air. When a hot bolt dropped on it, the robot stopped walking forward and self-gripped the bolt. Segment 4: The robot walked on a flat substrate in water. Segment 5: The robot curled its body in water in response to UV exposure.
